# Differential effects of synthetic estrogen on serum homocysteine levels before and after menopause

**DOI:** 10.1371/journal.pone.0338505

**Published:** 2025-12-10

**Authors:** Michael C. Reed, Ayako Suzuki, Allison Cruikshank, Mizuki Suzuki, H. Frederik Nijhout

**Affiliations:** 1 Department of Mathematics, Duke University, Durham, North Carolina, United States of America; 2 Department of Medicine, Duke University, Durham, North Carolina, United States of America; 3 Department of Medicine, Baylor University, Houston, Texas, United States of America; 4 Department of Biology, Duke University, Durham, North Carolina, United States of America; Belgrade University Faculty of Medicine, SERBIA

## Abstract

Homocysteine (Hcy), a sulfur-containing amino acid, is produced in prodigious quantities by the methionine cycle in the liver. Hcy is the major biomarker for cardio-vascular disorders and is associated with many other diseases. In previous work, we have explained why menstruating women have lower serum homocysteine than men due to higher concentrations of estradiol. In this study, we first present epidemiological evidence from NHANES data that synthetic estradiol supplementation lowers serum Hcy in post-menopausal women, but raises Hcy in pre-menopausal women. Secondly, we give an explanation of this puzzling phenomenon using previously developed mathematical models of one-carbon and glutathione metabolism. The simulation analysis demonstrated that the non-monotonic response of glutathione to rising estradiol levels may account for the differing Hcy responses to estradiol supplementation in postmenopausal versus premenopausal women, through activation of cystathionine β-synthase, a key enzyme regulating tissue homocysteine levels. Our findings further highlight the importance of considering menopausal status and synthetic hormone use when evaluating the health effects of homocysteine.

## Introduction

Homocysteine (Hcy) is a sulfur-containing amino acid produced via demethylating methionine, an essential amino acid derived from dietary protein. Since the late 1960s, the adverse health effects of elevated homocysteine have been recognized [1]. Elevated homocysteine contributes to cellular dysfunction and disease through several mechanisms; it increases oxidative stress and inflammation, damages endothelial cells, reduces nitric oxide availability, impairs vasodilation and enhances the conversion of homocysteine into highly reactive homocysteine thiolactone, which promotes the homocysteinylation of cellular proteins resulting in protein misfolding, aggregation, and loss of function, ultimately leading to cellular dyshomeostasis [2].

Elevated serum Hcy is associated with a wide range of disease conditions, including cardiovascular disease, atherosclerosis, cognitive dysfunction, cancers, and nonalcoholic fatty liver disease (NAFLD) [3–7]. Hcy is produced in the methionine cycle, where it can be remethylated to methionine, converted to thiolactone, or sent down the transsulfuration pathway toward glutathione by the enzyme cystathionine beta-synthase (CBS). These reactions are influenced by key nutrients such as vitamins B_6_, B_12_, B_9_ (folate), and betaine. Furthermore, the sex hormones influence the activity of many enzymes in folate, methionine and glutathione metabolism. This contributes to variations in serum Hcy levels within a population. For instance, serum homocysteine levels are typically higher in men than in women, in older individuals, and in postmenopausal than in premenopausal women [8,9]. Due to the many different effects of sex hormones on these complicated pathways, it is difficult to infer what the effects of supplemental estradiol will be.

This study has two parts. In the first part, we conduct an epidemiological analysis of synthetic hormone use on serum Hcy levels in pre-menopausal and post-menopausal women using NHANES data. Our analysis reveals differential effects of synthetic female sex hormones on serum Hcy levels in pre-menopausal versus post-menopausal women. Postmenopausal women using synthetic hormones had lower Hcy levels after use, while premenopausal women using synthetic hormones had higher Hcy levels after use.

In the second part, we use established mathematical models to explain the mechanistic reasons for these puzzling epidemiological results. Our mathematical models are based on the underlying physiology of the relevant biochemical pathways, assuming Michaelis-Menten kinetics with parameters determined from the literature. They have been validated by comparison with a large amount of experimental and clinical data. In previous work, we have explained why women have lower Hcy than men [10], the effects of oral contraceptives and B-vitamins on Hcy levels [11], and why women have higher glutathione than men [12]. In this study we use the mathematical model in Cruikshank et al. [12], with minor modifications to explain the epidemiological results above.

Because of the widespread use of synthetic estrogen, especially for women near and after menopause, it is important to women’s health to understand the effects of synthetic hormones on the underlying biochemical pathways in the body. In particular, understanding the impact of synthetic hormones on serum Hcy levels in women at different stages of the reproductive lifecycle is crucial for safety assessments, particularly in individuals with certain comorbidities. Our study contributes to the understanding of the effects of synthetic estrogen use on Hcy. We suggest that the effects of estrogen supplementation on other metabolites and pathways are an important field for future research.

## Results

### Clinical characteristics of the study population

Characteristics of the study population are summarized in Table 1. The mean age and BMI were 49 ± 20 years and 28.6 ± 7.1 kg/m^2^, respectively. Half of the female participants were postmenopausal at the time of the survey. Forty-eight percent were non-Hispanic White, 20% non-Hispanic Black, and 22.7% Mexican American. The mean serum homocysteine level was 8.3 ± 4.7 μmol/L. Fourteen percent of the female participants reported synthetic hormone use in the month prior to the survey date.

**Table 1:** The associations of population characteristics, serum homocysteine and serum cofactors with menopausal status.

	Summary statistics (N = 9,047)	Pre-menopausal (N = 4,505)	Post-menopausal (N = 4,542)	p-value#
Age, years	48.7 ± 20.0	31.9 ± 9.8	65.3 12.0	<0.0001
Race/ethnicity, %				<0.0001
Non-Hispanic White	48.2%	40.6%	55.7%	
Non-Hispanic Black	21.0%	23.9%	18.3%	
Mexican American	22.7%	26.5%	19.0%	
Other Hispanic	4.4%	5.0%	3.7%	
Other races/multiracial	3.6%	4.0%	3.3%	
BMI, kg/m2	28.6 ± 7.1	28.1 ± 7.4	29.1 ± 6.7	<0.0001
Serum homocysteine, μmol/L*	7.3 [6.0, 9.3]	6.4 [5.4, 7.6]	8.6 [7.0, 10.8]	<0.0001
Serum vitamin B12, pg/ml*	482 [359, 653]	463 [353, 611]	506 [364, 695]	<0.0001
Serum vitamin B6, nmol/L*	37.8 [21.1, 70.2]	33.6 [19.4, 56.9]	44.0 [23.2, 88.9]	<0.0001
Serum folate, ng/ml*	12.5 [8.9, 17.9]	11.0 [8.0, 14.7]	14.9 [10.2, 21.3]	<0.0001
MASLD, %**	58.9%	46.3%	71.1%	<0.0001
Post-menopausal women, %	50.2%	–	–	
Synthetic hormone use, %	14.1%	10.2%	17.9%	<0.0001
Estrogen alone	7.0%	1.4%	12.6%	
Progesterone alone	0.7%	0.9%	0.5%	
Combination	6.3%	7.9%	4.8%	

#: p-values from student-t tests for numeric variables and chi-square tests for categorical variables. *: Due to their skewed data distributions, data are presented as median [25^th^, 75^th^] and analyzed by Wilcoxon rank sum tests.

**: Metabolic dysfunction associated steatotic liver disease (MASLD) was defined by fatty liver index (FLI) of 30 or higher. Of note, 4,945 subjects (54.8%) had missing data required to compute the FLI.

There were significant differences in population characteristics, serum homocysteine levels, and serum cofactors between premenopausal and postmenopausal women, aside from age (Table 1). Premenopausal women had a higher prevalence of racial minorities, lower BMI, lower serum homocysteine levels, and lower serum cofactor levels compared to postmenopausal women. Postmenopausal women reported higher use of synthetic hormones, primarily estrogen alone, reflecting the common formula for hormone replacement therapy.

There were also missing data for serum vitamin measurements, including 123 for vitamin B12, 4,690 for vitamin B6, and 93 for folate.

Approximately 55% of the population had missing data required to compute the FLI and were excluded from the modeling analyses that adjusted for covariates. Comparisons of relevant characteristics between participants with and without FLI data are provided in S1 Table. The two populations are similar in demographic data, BMI, the prevalence of postmenopausal women, and the rate of synthetic hormone use; however, serum homocysteine and cofactor levels were statistically higher in the population without FLI (i.e., population excluded from multivariable analyses), though the differences were clinically insignificant.

### The associations between serum homocysteine and synthetic estrogens and progesterone use among adult women

The bivariate associations between serum homocysteine levels and synthetic hormone use categories are summarized in Table 2. In the overall population, there was a significant association between serum homocysteine levels and synthetic hormone use categories (p < 0.0001). Interestingly, the observed associations differed between pre- and postmenopausal women (p = 0.362 and p < 0.0001, respectively). Among postmenopausal women, those not using synthetic hormones had the highest serum homocysteine levels, whereas, in premenopausal women, those not using synthetic hormones had lower serum homocysteine levels compared to women using estrogen alone.

**Table 2:** The bivariate association between serum homocysteine levels and synthetic hormone use.

	Overall		Premenopausal women		Postmenopausal women	
	N	Serum Hcy	N	Serum Hcy	N	Serum Hcy
*Synthetic hormone use*		P < 0.0001		P = 0.362		P < 0.0001
None	7776	7.3 [6.0, 9.4]	4045	6.4 [5.4, 7.6]	3731	8.9 [7.2, 11.2]
Estrogen alone	637	7.5 [6.4, 9.3]	64	6.7 [5.5, 8.0]	573	7.6 [6.5, 9.4]
Progesterone alone	62	6.7 [5.5, 9.0]	41	6.5 [5.3, 7.9]	21	7.4 [5.8, 9.8]
Combination	572	6.7 [5.7, 8.0]	355	6.3 [5.4, 7.3]	217	7.4 [6.4, 8.9]

To further characterize the associations and potential effect modification by menopausal status, we performed multiple linear regression models adjusting for potential confounders. The results of the multiple linear regression models, both with and without adjustment for confounders, are summarized in Table 3. Due to significant missing FLI data, all modeling analyses were performed on the subpopulation with available FLI data (N = 4,093). After adjusting for age, race/ethnicity, and MASLD status (i.e., FLI category), synthetic estrogen use was differentially associated with serum homocysteine levels in pre- and postmenopausal women. Among premenopausal women, those using estrogen alone had higher serum homocysteine levels, whereas postmenopausal women using estrogen alone had lower serum homocysteine levels compared to those not using synthetic hormones. Specifically, premenopausal women using estrogen alone had a higher serum homocysteine by an average of 1.15 ± 0.53 μmol/L compared to serum homocysteine in those not using synthetic hormones, while postmenopausal women using estrogen had a lower serum homocysteine by an average of −0.86 ± 0.33 μmol/L. Progesterone alone appears to have no significant effect on serum homocysteine levels, regardless of menopausal status. However, postmenopausal women using combined hormones showed a much lower serum homocysteine (−1.33 ± 0.50 μmol/L) compared to those not using synthetic hormones, a difference larger than that observed for estrogen alone (−0.86 ± 0.33 μmol/L), suggesting a potential synergistic effect.

**Table 3:** The association between serum homocysteine levels and synthetic hormone use with and without adjusting for other confounders in multiple linear regression models.

	N	Unadjusted		Adjusted	
		β ± SE	P-value	β ± SE	P-value
**Premenopausal women**					
*Synthetic hormone use*					
None	1808	–		–	
Estrogen alone	30	0.59 ± 0.44	0.176	1.15 ± 0.53	0.028
Progesterone alone	17	0.06 ± 0.54	0.909	0.27 ± 0.69	0.698
Combination	161	−0.41 ± 0.19	0.031	−0.35 ± 0.24	0.147
**Postmenopausal women**					
*Synthetic hormone use*					
None	1673	–		–	
Estrogen alone	276	−1.49 ± 0.23	<0.0001	−0.86 ± 0.33	0.0096
Progesterone alone	15	−1.62 ± 1.14	0.155	−0.35 ± 1.31	0.787
Combination	113	−2.06 ± 0.36	<0.0001	−1.33 ± 0.50	0.0076

*: the multiple linear regression models were adjusted for age, race/ethnicity, and FLI categories (MASLD yes vs. no).

### Simulation analyses: investigating the dichotomy observed in the association between serum homocysteine levels and synthetic hormone use in pre- and post-menopausal women

**Fig 1:**
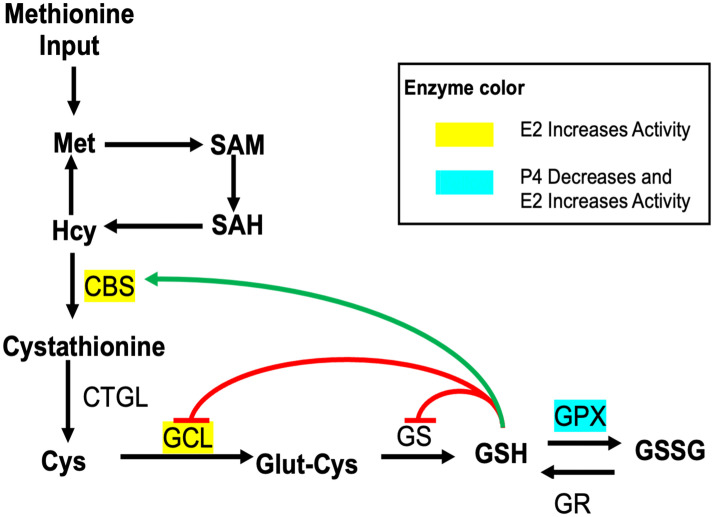
Regulatory reaction diagram for glutathione and one-carbon metabolism. Substrates are indicated by bold lettering. Each black arrow represents a biochemical reaction and next to some are the acronyms of the enzymes that catalyze the reactions. The yellow highlighted names indicate the enzymes that are upregulated by estradiol and the blue highlighted name indicates that GPX is upregulated by estradiol and downregulated by progesterone. The green arrow from GSH signifies that GSH stimulates CBS. The red arrows from GSH signify that GSH inhibits GCL and GS. Many reactions and mechanisms that are present in the mathematical model are not shown in this simplified diagram. Substrate abbreviations: Met, methionine; SAM, S-adenosylmethionine; SAH, S-adenosylhomocysteine; Hcy, homocysteine; Cys, Cysteine; Glut-cys, glutamyl-cysteine; GSH, glutathione; GSSG, glutathione disulfide; E2, estradiol; P4, progesterone. Enzyme Abbreviations: CBS, cystathionine β-synthase; CTGL, γ-cystathionase; GCL, γ-glutamylcysteine synthetase; GS, glutathione synthetase; GPX, glutathione peroxidase; GR, glutathione reductase.

Using our mathematical model, we explain why estrogen supplementation increases homocysteine levels in premenopausal women but decreases them in postmenopausal women as seen in the data analysis above. Fig 1 shows a simplified regulatory diagram of the methionine cycle and the glutathione pathway. It should be noted that the other outputs of the methionine cycle including the polyamine pathway, and various other substrates/enzymes that are included in our mathematical model are not shown here. Only the elements that are relevant to the explanation of our results are shown in Fig 1. Importantly, progesterone inhibits, and estradiol stimulates GPX activity and estradiol also stimulates GCL and CBS. In addition, GSH stimulates CBS activity and inhibits its own production by decreasing the activity of GS and GCL. Despite these complications, the mathematical model allows us to understand the effects of estrogen supplementation.

**Fig 2:**
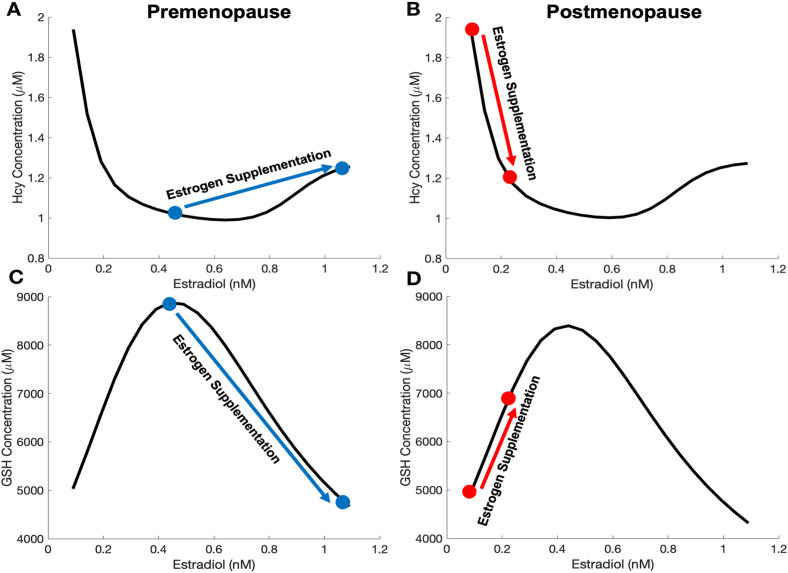
Hcy and GSH with estradiol supplementation in premenopausal and postmenopausal women. Model predictions of Hcy variation as estradiol increases in premenopausal and postmenopausal women are plotted in panels A and B, respectively. Model predictions of GSH variation as estradiol increases in premenopausal and postmenopausal women are plotted in panels C and D, respectively. Estrogen supplementation is indicated by the colored arrows in each figure. See the Methods for a discussion of the values for estradiol chosen to represent estradiol supplementation. The black GSH curve in panels C and D comes from [12].

The changes in Hcy and GSH concentrations after estradiol supplementation in premenopausal and postmenopausal women are shown in Fig 2. Notice that the GSH curve in panels C and D does not monotonically increase with estradiol. Instead, GSH increases until it reaches a maximum and then decreases. To understand this non-monotone behavior of GSH, it is crucial to consider that there are two incoming fluxes to GSH, GS and GR, and two outgoing fluxes, GPX and a large flux out into the blood (not shown in Fig 1). At steady state, the incoming fluxes must balance the outgoing fluxes. When estradiol is low, the GS flux rises more rapidly than the GPX flux as estradiol increases, pushing GSH up. Then, as GSH starts to inhibit its own synthesis, GS flux increases more slowly while the GPX flux continues to increase at roughly in proportion to estradiol, so the GSH curve starts to level off and eventually reaches a peak. Then, as estradiol levels increase even further, the increased GPX flux dominates as the GS flux increases ever more slowly and eventually declines. This behavior of the GS flux is caused by the competition between the higher inhibition of GS and GCL by GSH and the higher stimulation of GCL by estradiol. The Hcy level depends on the level of GSH since GSH activates CBS (see Fig 1). Now, at steady state the flux into the methionine cycle must approximately equal the flux out via CBS, since the polyamine pathway and other outputs drain little mass from the cycle relative to CBS. So, when GSH increases and stimulates CBS, Hcy decreases, which allows the CBS flux to remain unchanged. Similarly, when GSH decreases and the stimulation of CBS decreases, Hcy increases to keep the CBS flux unchanged. This relationship explains the shape of the GSH and Hcy curves as estradiol levels rise. The average estradiol concentration is 0.4368 nM in premenopausal women and the GSH level is near the maximum of the GSH curve. Thus, estradiol supplementation causes GSH to decrease and Hcy to increase in premenopausal women. Conversely, since the average estradiol concentration is 0.09 nM in postmenopausal women, the GSH level is very low (see panel D in Fig 2). Thus, estradiol supplementation causes GSH to increase and therefore Hcy to decrease in postmenopausal women as discussed in the previous section and shown in Tables 2 and 3.

**Fig 3:**
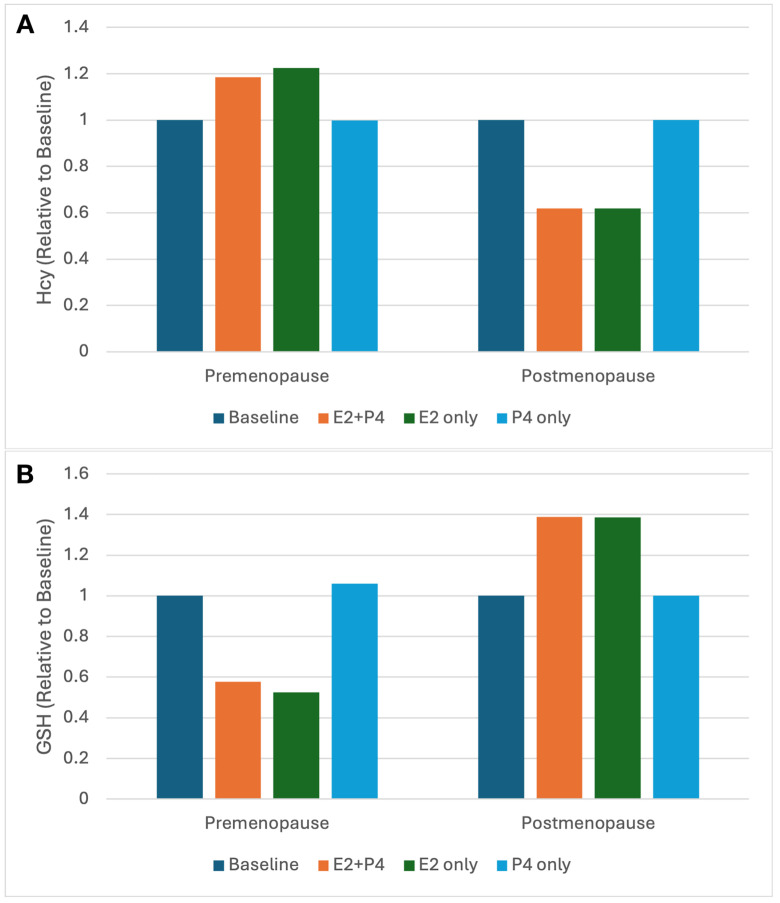
GSH and Hcy with various hormone therapies. Model predictions of relative Hcy with/without estradiol and/or progesterone therapy in premenopausal and postmenopausal women are plotted in panel **A.** Model predictions of relative GSH with/without estradiol and/or progesterone therapy in premenopausal and postmenopausal women are plotted in panel **B.** See the Methods for a discussion of the values for estradiol and progesterone chosen to represent different hormone therapies.

Fig 3 shows the relative Hcy and GSH levels, computed with the mathematical model, in premenopausal and postmenopausal women without any hormone therapy, and with estradiol and/or progesterone supplementation. In premenopausal women, estradiol or estradiol + progesterone supplementation leads to an increase in Hcy and a decrease in GSH. In postmenopausal women, the same supplementation causes a decrease in Hcy and an increase in GSH. Following the above reasoning, this can be attributed to the fact that GSH levels do not increase monotonically with estradiol; instead, GSH rises to a peak and then declines as estradiol continues to increase. Since GSH stimulates CBS, as we explained above, this results in an opposite effect on Hcy. Additionally, since progesterone does not significantly alter GPX activity, we see the same results in estradiol + progesterone supplementation as with estradiol supplementation only. It’s important to note that progesterone supplementation alone does not significantly affect GSH or Hcy levels in either group, as progesterone does not substantially influence GPX activity. This is consistent with the statistical data on the effects of progesterone supplementation reported above in the previous section (see Tables 2 and 3).

This analysis draws on two key concepts discussed in previous papers [10–14]. First, the flux out of the methionine cycle via CBS is approximately equal to the input of methionine from the blood. This is because the other outputs of the methionine cycle, e.g., the polyamine pathway, extract relatively little mass compared to CBS. Therefore, if CBS is activated (e.g., by GSH), at steady state the homocysteine concentration will drop so that the flux through CBS remains approximately the same. Conversely, if CBS is inhibited then, at steady state, the homocysteine concentration will rise so that the CBS flux remains approximately the same. The second key concept, demonstrated in [12], is that an increase in estrogen from typical low male levels to average female levels causes an increase in the level of glutathione. However, above a certain threshold, increasing estradiol actually decreases glutathione levels because of the feedback inhibition on its own synthesis. Both concepts are crucial for understanding why estrogen supplementation raises Hcy for premenopausal women and lowers Hcy for postmenopausal women.

## Discussion

Using data from a large general population survey, we demonstrated that synthetic hormone use was significantly associated with serum homocysteine levels in a menopausal-state-specific manner. Specifically, premenopausal women using synthetic estrogen alone had higher serum homocysteine levels compared to those not using synthetic hormones, whereas postmenopausal women using synthetic estrogen alone had lower levels. This suggests that the effect of estrogen on serum homocysteine levels varies depending on menopausal status. Progesterone alone had no significant effect on serum homocysteine levels, regardless of menopausal status.

Our mathematical model reveals the mechanism behind the differences in Hcy levels in pre- and post-menopausal women following estrogen supplementation. Specifically, we find that variations in GSH levels drive the differences in the responses of Hcy to estradiol supplementation in these groups. As estrogen levels rise, GSH increases to a peak and then declines. This pattern is significant because GSH activates CBS, the enzyme that sends Hcy down the transsulfuration pathway. Since CBS flux is approximately constant, its activation must lower Hcy levels, and CBS inhibition must raise Hcy levels. In pre-menopausal women, where baseline estrogen is higher, additional estrogen reduces GSH and raises Hcy levels. Conversely, in post-menopausal women with lower baseline estrogen, supplementation raises GSH levels, leading to a decrease in Hcy.

Estrogens exert a wide range of effects in humans. Notably, synthetic estrogens have been linked to vitamin B6 metabolism and altered tryptophan metabolism, which relies on vitamin B6 [15]. Existing epidemiological data suggest that current low-dose oral contraceptives (Ocs) may negatively affect vitamin B6 status [16]. Additionally, a previous clinical study [17] showed that oral estradiol reduced both plasma homocysteine and vitamin B6 levels in postmenopausal women. In our extended analysis using NHANES data, the inclusion of serum vitamin B_6_ in the model changed the effect size of synthetic estrogens on serum homocysteine levels—reducing the effect in premenopausal women and increasing it in postmenopausal women by approximately 15%. This indicates that about 15% of the estrogen-induced rise in serum homocysteine in premenopausal women may be explained by the interaction between estrogen and vitamin B_6_. Similarly, around 15% of the estrogen-driven reduction in homocysteine in postmenopausal women may be influenced by this interplay. Further studies are needed to confirm this hypothesis.

Despite the elevated serum homocysteine levels among premenopausal women, the clinical significance of this elevation remains uncertain. Notably, serum homocysteine levels were significantly higher in postmenopausal than in premenopausal women, regardless of estrogen use (data not shown). Previous analyses demonstrated that higher serum homocysteine was associated with increased hepatic fibrosis risk among MASLD patients, but only in men and postmenopausal women, not premenopausal women [18]. Our extended NHANES analysis showed that a history of cardiovascular disease was more common among postmenopausal women, with a lower prevalence among those using estrogens, whereas no association between estrogen use and cardiovascular disease history was observed among premenopausal women (data not shown). These findings suggest that the health impact of elevated homocysteine and synthetic estrogen use may differ by age, sex, menopausal status, or target organ, and further studies incorporating these factors are warranted.

Our study has several limitations that warrant cautious interpretation of the data. First, our epidemiological analysis was cross-sectional, based on existing US general population data from NHANES. As such, it addresses associations between outcomes and predictors at the time of the survey but does not establish causality. While the observed effect of estrogens on serum homocysteine levels in postmenopausal women has been validated in a clinical trial [17], this has not been investigated among premenopausal women. Based on our simulation analysis, further studies are warranted to investigate the effects of estrogens on serum homocysteine in premenopausal women, accounting for different doses and interactions with serum vitamin B_6_. Additionally, to adjust for the potential contribution of MASLD to serum homocysteine levels, we included a variable created using the FLI, an established measure incorporating BMI, waist circumference, serum triglycerides, and serum GGT [19]. However, no direct assessment of hepatic fat accumulation or diagnosis was available in our analysis, which may have introduced misclassification. Our mathematical model is based on the underlying physiology, but no model can encompass the entirety of biological complexity. The parameters and kinetic constants in our model come from experimental data in the literature when present. Even so, the mathematical model incorporates simplifications. The amount of estradiol produced during the menstrual cycle varies a lot in different women. In our statistical analysis and mathematical model, we assume a fixed estrogen level for simplicity as estrogen status is unknown in the NHANES study.

Despite the above limitations, this study has several important clinical and scientific implications. First, although hormone replacement therapy is linked to lower serum homocysteine levels in postmenopausal women, our NHANES data analysis is the first to show that synthetic estrogens may raise serum (and possibly tissue) homocysteine levels in premenopausal women, suggesting potential negative effects for women with elevated homocysteine conditions. Second, using a mathematical model for GSH metabolism, we showed that the effects of supplemental estrogen on pre- and post-menopausal women could be explained by the activation of CBS by GSH and the nonmonotonic behavior of the GSH curve as a function of estrogen. For the full model, see Cruikshank et al. [12] and the methods of this paper. Such interdisciplinary efforts may facilitate scientific discovery and aid in establishing individualized patient care by addressing key puzzling biological observations. Lastly, our findings underscore the importance of considering reproductive status in biomedical research. The need to account for sex differences and variations in physiological hormone levels due to reproductive status, genetic variants, and pathological conditions has become increasingly recognized. Given the strong regulation of one-carbon metabolism by sex hormones, future research on homocysteine metabolism should be designed with these factors in mind.

## Methods

### Epidemiological analysis

#### Data source and study design.

We utilized data from the National Health and Nutrition Examination Survey (NHANES) for the years 1999–2006, during which serum homocysteine measurements were available for participants. We conducted a cross-sectional analysis to examine the association between serum homocysteine levels and self-reported use of synthetic female sex hormones (e.g., estrogen and progesterone) at the time of the survey. Female respondents were classified into premenopausal and postmenopausal for this analysis.

#### Study population.

**Fig 4:**
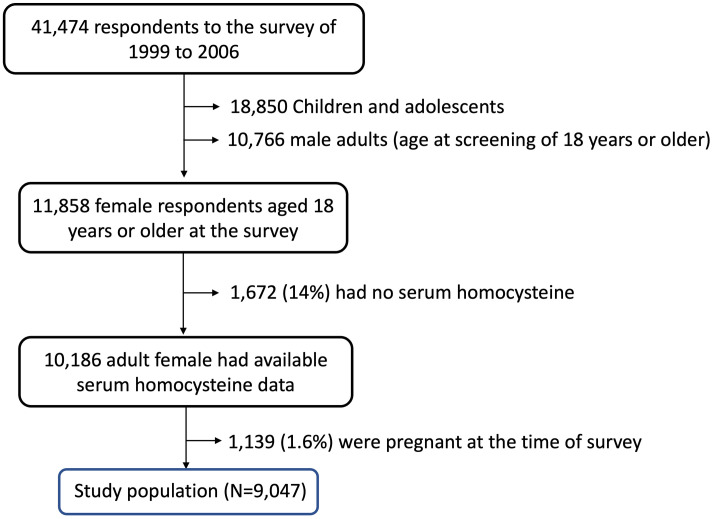
The algorithm to define the study population.

Among a total of 41,474 respondents to the survey conducted between 1999 and 2006, we selected 11,858 female subjects aged 18 or older, of whom 10,186 had available serum homocysteine data. Female participants who were pregnant at the time of the survey were excluded. The final study cohort included 9,047 female participants, who comprised our study population (Fig 4).

Serum homocysteine data were available from survey records between 1999 and 2006, which defined the source of the study population. After excluding children, adolescents, adult males, participants without available serum homocysteine levels, and women who were pregnant at the time of the survey, we identified 9,047 adult female participants as our study population.

### Classification of menopausal status and synthetic hormone use

The study cohort was further divided into premenopausal and postmenopausal women based on self-reported reproductive information or a history of bilateral oophorectomy. In cases where no menopausal information was available, the age 51 years, an average age at menopause in the United States [20], were used as the cutoff between pre-and postmenopausal women. Synthetic hormone users were identified using medication tables, which contains all the medications taken during a one-month prior to the survey date. Participants were classified into four categories: estrogen alone, progesterone alone, estrogen/progesterone combination, and no hormone use for the analysis.

### Other study variables

Demographic data, BMI, serum homocysteine, serum vitamin B_12_, B_6_, and folates were retrieved from the NHANES database for our analysis. Serum homocysteine levels have been associated with metabolic dysfunction-associated steatotic liver disease (MASLD), previously known as nonalcoholic fatty liver disease (NAFLD)v[6]. Given that approximately one-third of the adult population in the U.S. is affected by this condition [21,22], we computed a fatty liver index (FLI) [19] and classified the population as either MASLD-negative (defined as probability <30%) or MASLD-positive or indeterminate (probability of>=30%) using a cut-off value of 30, which was considered as a covariate in the analysis.

### Statistical analyses

Data are presented as mean ± SD for continuous variables and percentage for categorical/ranked variables. The primary study outcome was serum homocysteine, and synthetic hormone use was the primary predictor in the analysis. The associations between serum homocysteine levels and synthetic hormone use (estrogen alone, progesterone alone, estrogen/progesterone combination, and no hormone use) were analyzed in the overall population and in the subgroups by menopausal status, using ANOVA and multiple linear regression models, adjusting for relevant covariates. All the statistical analyses were performed using JMP® Pro (version 17.2.0, SAS, Cary, NC).

### Human Subjects

The DUHS IRB Board declared this study exempt from IRB review.

### Simulation analysis

#### Mathematical model.

To evaluate the impact of synthetic hormones on serum homocysteine levels among pre- and post-menopausal women, we used mathematical models simulating the complex regulation of one-carbon metabolism and its peripheral pathways, developed by Michael Reed, H. Frederik Nijhout, and Allison Cruikshank [10–12]. For this particular study, we use the mathematical model in Cruikshank et al. [12] so that we can calculate Hcy concentrations in response to various levels of estrogen and progesterone.

#### Progesterone and estradiol levels.

In our model, the estradiol concentration is 0.4368 nM for premenopausal women and 0.09 nM for postmenopausal females [23]. The progesterone levels during a 28 day menstrual cycle are taken from [24]. To obtain the average progesterone level, we first used Matlab’s spline function to obtain a time dependent progesterone curve and then calculated the average value of that curve. The progesterone concentration for the average woman is 12.5257 nM. For postmenopausal women, we take the average progesterone concentration to be 0.318 nM [25].

#### Progesterone and Estradiol Effect on GPX.

Fig 1 shows a simplified diagram of the full model. Multiple studies have shown that progesterone decreases GPX activity or GPX enzyme concentrations *in vitro* [26–28]. In [26], rat hepatic stellate cells were treated with estradiol (E2) or progesterone (P4) for 24 hours *in vitro*, and found that progesterone had a smaller effect on GPX activity than estradiol. For example, a 100 nM dose of estradiol increased GPX activity by 140% and a 100 nM dose of progesterone deceased the activity of GPX by 25%. Thus, in our model the strength of estradiol activation on GPX is larger than the progesterone inhibition on GPX. In [12], we used a linear increasing function of estradiol for the effect of estradiol on GPX. To make this model more biologically realistic, we here use a Michaelis-Menten function that saturates at high estradiol levels. Taken from the experimental literature, the parameter values were fitted to represent an 80% increase in activity between males and females.


$$\;\text{E}2\;\text{activation}\;\text{of}\;\text{GPX}\;(\text{multiplies}\;\text{Vmax}):\;1\;+\;\frac{27(E2-0.09)}{11\;+\;(E2-0.09)\;}\;(\text{E}2\;\text{is}\;\text{in}\;\text{nM})$$

$$\text{P}4\;\text{inhibition}\;\text{of}\;\text{GPX}\;(\text{multiplies}\;\text{Vmax}):\;1\;-\;\frac{0.3(P4-0.318)}{70\;+\;(P4-.318)}\;(\text{P}4\;\text{is}\;\text{in}\;\text{nM})$$

#### E2 Effect on GCL.

In [12], we used a linear increasing function of estradiol for the effect of estradiol on GCL. To make this model more physiological, we have changed this function to be a Michaelis-Menten function such that it saturates at high estradiol levels. We chose the parameter values that represent an 80% increase in activity between males and females as is the consensus in the literature.


$$\;\text{E}2\;\text{activation}\;\text{of}\;\text{GCL}\;(\text{multiplies}\;\text{Vmax}):\;1\;+\;\frac{25.77(E2-0.09)}{10.8\;+\;(E2-0.09)}\;(\text{E}2\;\text{is}\;\text{in}\;\text{nM})$$

#### GSH Effect on CBS.

The authors in [29] found that glutathionylation of CBS increases its activity. Thus, we added the activation of glutathione via CBS. It takes a Michaelis-Menten function form:


$$\;\text{GSH}\;\text{forcing}\;\text{on}\;\text{CBS}\;(\text{multiplies}\;\text{Vmax}):\;1+\frac{V_{max}*cgsh^{H}}{Km_{gsh}^{H}+cgsh^{H}},$$

where V_max_ =.4, K_m_ = 6000, H = 10.

#### Estrogen and Progesterone Supplementation.

Estrogen and progesterone supplementation is assumed to increase estradiol and progesterone levels by a factor 1.5. There are numerous papers that measure serum hormone levels after different kinds of hormone therapy in premenopausal and postmenopausal women [30–33]. Since we did not know the dose or the method of hormone therapy in the women in those studies, we chose a 150% increase that represents an average of the data in the literature for supplementation. Thus, for postmenopausal females, the baseline estradiol concentration is 0.09 nM and estradiol supplementation increases these levels 1.5-fold to 0.2250 nM. The baseline progesterone concentration is 0.318 nM and progesterone supplementation increases this concentration 1.5-fold to 0.7950 nM. Similarly, for premenopausal females, the baseline estradiol concentration is 0.4368 nM and estradiol supplementation increases this concentration 1.5-fold to 1.0920 nM. The baseline progesterone concentration is 12.5257 nM and progesterone supplementation increases this concentration 1.5-fold to 31.3143 nM.

## Supporting information

S1 Table Supplemental Table. There were missing data: Vitamin B6 (N = 4690), vitamin B12 (N = 123), and serum folate (N = 93). Serum vitamin B6 was only available from the survey 2003–2004.
(DOCX)

S2 Code Model Code. (PDF)

S3 Data NHANES Data. (XLSX)

## References

[1] McCully KS. Vascular pathology of homocysteinemia: implications for the pathogenesis of arteriosclerosis. Am J Pathol. 1969;56(1):111–28. 5792556 PMC2013581

[2] Perła-Kaján J, Twardowski T, Jakubowski H. Mechanisms of homocysteine toxicity in humans. Amino Acids. 2007;32(4):561–72. doi: 10.1007/s00726-006-0432-9 17285228

[3] Homocysteine Studies Collaboration. Homocysteine and risk of ischemic heart disease and stroke: a meta-analysis. JAMA. 2002;288(16):2015–22. doi: 10.1001/jama.288.16.2015 12387654

[4] Seshadri S, Beiser A, Selhub J, Jacques PF, Rosenberg IH, D’Agostino RB, et al. Plasma homocysteine as a risk factor for dementia and Alzheimer’s disease. N Engl J Med. 2002;346(7):476–83. doi: 10.1056/NEJMoa011613 11844848

[5] Zhang D, Wen X, Wu W, Guo Y, Cui W. Elevated homocysteine level and folate deficiency associated with increased overall risk of carcinogenesis: meta-analysis of 83 case-control studies involving 35,758 individuals. PLoS One. 2015;10(5):e0123423. doi: 10.1371/journal.pone.0123423 25985325 PMC4436268

[6] Yuan S, Chen J, Dan L, Xie Y, Sun Y, Li X, et al. Homocysteine, folate, and nonalcoholic fatty liver disease: a systematic review with meta-analysis and Mendelian randomization investigation. Am J Clin Nutr. 2022;116(6):1595–609. doi: 10.1093/ajcn/nqac285 36205540

[7] Tripathi M, Singh BK, Zhou J, Tikno K, Widjaja A, Sandireddy R, et al. Vitamin B12 and folate decrease inflammation and fibrosis in NASH by preventing syntaxin 17 homocysteinylation. J Hepatol. 2022;77(5):1246–55. doi: 10.1016/j.jhep.2022.06.033 35820507

[8] Xu R, Huang F, Wang Y, Liu Q, Lv Y, Zhang Q. Gender- and age-related differences in homocysteine concentration: a cross-sectional study of the general population of China. Sci Rep. 2020;10(1):17401. doi: 10.1038/s41598-020-74596-7 33060744 PMC7566483

[9] Zhu Z, Jiang S, Li C, Liu J, Tao M. Relationship between serum homocysteine and different menopausal stage. Climacteric. 2020;23(1):59–64. doi: 10.1080/13697137.2019.1634045 31294633

[10] Sadre-Marandi F, Dahdoul T, Reed MC, Nijhout HF. Sex differences in hepatic one-carbon metabolism. BMC Syst Biol. 2018;12(1):89. doi: 10.1186/s12918-018-0621-7 30355281 PMC6201565

[11] Kim R, Nijhout HF, Reed MC. One-carbon metabolism during the menstrual cycle and pregnancy. PLoS Comput Biol. 2021;17(12):e1009708. doi: 10.1371/journal.pcbi.1009708 34914693 PMC8741061

[12] Cruikshank A, Reed MC, Nijhout HF. Sex differences in glutathione metabolism and acetaminophen toxicity. Metab Target Organ Damage. 2024;4(2). doi: 10.20517/mtod.2023.44

[13] Reed MC, Gamble MV, Hall MN, Nijhout HF. Mathematical analysis of the regulation of competing methyltransferases. BMC Syst Biol. 2015;9:69. doi: 10.1186/s12918-015-0215-6 26467983 PMC4606511

[14] Nijhout HF, Reed MC, Budu P, Ulrich CM. A mathematical model of the folate cycle: new insights into folate homeostasis. J Biol Chem. 2004;279. 10.1074/jbc.M410818200 15496403

[15] Wilson SMC, Bivins BN, Russell KA, Bailey LB. Oral contraceptive use: impact on folate, vitamin B₆, and vitamin B₁₂ status. Nutr Rev. 2011;69(10):572–83. doi: 10.1111/j.1753-4887.2011.00419.x 21967158

[16] Lussana F, Zighetti ML, Bucciarelli P, Cugno M, Cattaneo M. Blood levels of homocysteine, folate, vitamin B6 and B12 in women using oral contraceptives compared to non-users. Thromb Res. 2003;112(1–2):37–41. doi: 10.1016/j.thromres.2003.11.007 15013271

[17] Smolders RGV, de Meer K, Kenemans P, Jakobs C, Kulik W, van der Mooren MJ. Oral estradiol decreases plasma homocysteine, vitamin B6, and albumin in postmenopausal women but does not change the whole-body homocysteine remethylation and transmethylation flux. J Clin Endocrinol Metab. 2005;90(4):2218–24. doi: 10.1210/jc.2004-1021 15671107

[18] Suzuki M, Kim HY, Reed MC, Nijhout HF, Cruikshank A, Abdelmalek M, et al. Elevated Homocysteine is Associated With Liver Fibrosis in Metabolic Dysfunction-Associated Steatotic Liver Disease in a Sex- and Menopause-Specific Manner. Gastro Hep Adv. 2025;5(1):100800. doi: 10.1016/j.gastha.2025.100800 41211504 PMC12589971

[19] Bedogni G, Bellentani S, Miglioli L, Masutti F, Passalacqua M, Castiglione A, et al. The Fatty Liver Index: a simple and accurate predictor of hepatic steatosis in the general population. BMC Gastroenterol. 2006;6:33. doi: 10.1186/1471-230X-6-33 17081293 PMC1636651

[20] Freedman DM, Tarone RE. Re: “From menarche to menopause: trends among US women born from 1912 to 1969”. Am J Epidemiol. 2007;166(7):860–1. doi: 10.1093/aje/kwm228 17698972

[21] Browning JD, Szczepaniak LS, Dobbins R, Nuremberg P, Horton JD, Cohen JC, et al. Prevalence of hepatic steatosis in an urban population in the United States: impact of ethnicity. Hepatology. 2004;40(6):1387–95. doi: 10.1002/hep.20466 15565570

[22] Szczepaniak LS, Nurenberg P, Leonard D, Browning JD, Reingold JS, Grundy S, et al. Magnetic resonance spectroscopy to measure hepatic triglyceride content: prevalence of hepatic steatosis in the general population. Am J Physiol Endocrinol Metab. 2005;288(2):E462-8. doi: 10.1152/ajpendo.00064.2004 15339742

[23] ARCHITECT. Estradiol. 2009.

[24] Stricker R, Eberhart R, Chevailler M-C, Quinn FA, Bischof P, Stricker R. Establishment of detailed reference values for luteinizing hormone, follicle stimulating hormone, estradiol, and progesterone during different phases of the menstrual cycle on the Abbott ARCHITECT analyzer. Clin Chem Lab Med. 2006;44(7):883–7. doi: 10.1515/CCLM.2006.160 16776638

[25] ARCHITECT Progesterone. 2010.

[26] Itagaki T, Shimizu I, Cheng X, Yuan Y, Oshio A, Tamaki K, et al. Opposing effects of oestradiol and progesterone on intracellular pathways and activation processes in the oxidative stress induced activation of cultured rat hepatic stellate cells. Gut. 2005;54(12):1782–9. doi: 10.1136/gut.2005.053278 16284289 PMC1774809

[27] Ohwada M, Suzuki M, Sato I, Tsukamoto H, Watanabe K. Glutathione peroxidase activity in endometrium: effects of sex hormones and cancer. Gynecol Oncol. 1996;60(2):277–82. doi: 10.1006/gyno.1996.0038 8631551

[28] Yuan X-H, Fan Y-Y, Yang C-R, Gao X-R, Zhang L-L, Hu Y, et al. Progesterone amplifies oxidative stress signal and promotes NO production via H2O2 in mouse kidney arterial endothelial cells. J Steroid Biochem Mol Biol. 2016;155(Pt A):104–11. doi: 10.1016/j.jsbmb.2015.09.029 26462682

[29] Niu WN, Yadav PK, Adamec J, Banerjee R. S-glutathionylation enhances human cystathionine B-synthase activity under oxidative stress conditions. Antioxidants and Redox Signaling. 2015;22(5). 10.1089/ars.2014.5891 PMC4307034 24893130

[30] Sriprasert I, Hodis HN, Bernick B, Mirkin S, Mack WJ. Effects of Estradiol Dose and Serum Estradiol Levels on Metabolic Measures in Early and Late Postmenopausal Women in the REPLENISH Trial. J Womens Health (Larchmt). 2020;29(8):1052–8. doi: 10.1089/jwh.2019.8238 32644875 PMC7759286

[31] Kim SM, Kim SE, Lee DU, Choi D. Serum estradiol level according to dose and formulation of oral estrogens in postmenopausal women. Nature Research. 2021;11. 10.1038/s41598-021-81201-y PMC7878477 33574350

[32] Andréen L, Bixo M, Nyberg S, Sundström-Poromaa I, Bäckström T. Progesterone effects during sequential hormone replacement therapy. Eur J Endocrinol. 2003;148(5):571–7. doi: 10.1530/eje.0.1480571 12720542

[33] Hamada AL, Maruo T, Samoto T, Yoshida S, Nash H, Spitz IM, et al. Estradiol/progesterone-releasing vaginal rings for hormone replacement therapy in postmenopausal women. Gynecological Endocrinology. 2003;17(3):247–54. doi: 10.1080/gye.17.3.247.254 12857433

