# Supplementary material for: Differential effects of synthetic estrogen on serum homocysteine levels before and after menopause
Source: PLoS One. 2025 Dec 10;20(12):e0338505. doi: 10.1371/journal.pone.0338505 (PMC12694790; doi:10.1371/journal.pone.0338505)
Supplement: S1 Table — There were missing data: Vitamin B6 (N = 4690), vitamin B12 (N = 123), and serum folate (N = 93). Serum vitamin B6 was only available from the survey 2003–2004. (DOCX) [file pone.0338505.s001.docx]

**Supplemental table**

|  | Without FLI  (N=4,954) | With FLI  (N=4,093) | p-value# |
| --- | --- | --- | --- |
| Age, years | 48.9 ± 20.3 | 48.5 ± 19.7 | 0.312 |
| Race/ethnicity, % |  |  | 0.839 |
| Non-Hispanic White | 48.0% | 48.4% |  |
| Non-Hispanic Black | 20.9% | 21.4% |  |
| Mexican American | 22.9% | 22.6% |  |
| Other Hispanic | 4.5% | 4.2% |  |
| Other races/multiracial | 3.8% | 3.5% |  |
| BMI, kg/m2 | 28.6 ± 7.1 | 28.6 ± 7.0 | 0.726 |
| Serum homocysteine, **μmol/L*** | 7.5 [6.1, 9.5] | 7.2 [5.9, 9.0] | <0.0001 |
| Serum vitamin B12, pg/ml* | 487 [361, 661] | 476 [358, 644] | 0.083 |
| Serum vitamin B6, nmol/L* | 38.2 [21.6, 72.5] | 36.8 [20.4, 66.3] | 0.033 |
| Serum folate, ng/ml* | 12.7 [9.0, 18.2] | 12.2 [8.7, 17.6] | 0.003 |
|  | 50.2% | 50.7% | 0.3498 |
| Postmenopausal women, % |  |  |  |
| Synthetic hormone use, % | 13.3% | 15.0% | 0.148 |
| Estrogen alone | 6.7% | 7.5% |  |
| Progesterone alone | 0.6% | 0.8% |  |
| Combination | 6.0% | 6.7% |  |
