# Supplementary material for: Differential effects of synthetic estrogen on serum homocysteine levels before and after menopause
Source: PLoS One. 2025 Dec 10;20(12):e0338505. doi: 10.1371/journal.pone.0338505 (PMC12694790; doi:10.1371/journal.pone.0338505)
Supplement: S2 Code — (PDF) [file pone.0338505.s002.pdf]

```
% Differential Effects of Synthetic Estrogen on Homocysteine in Pre- and
% Post- menopausal women
% 2024 Reed, Suzuki, Cruikshank, Suzki, Nijhout
% MATLAB code

%% steady state simulations
p = parameters;

%%% Change e2 and p4 depending on menopausal status & HRT used %%%
p.val(p.e2) = .4368;
p.val(p.p4) = 12.5257;
%%%%%%%%%%%%%%%%%%%%%%%%%

ode_run(p,'premeno_NoHRT'); % saves matfile with steady state model
values for premenopausal women without HRT


%% run simulations for predefined parameter structure p

function ode_run(p,type)
% saves model concentrations and velocities in a matfile

H2O2 = (1)*0.01; % change for oxidative stress experiments
ssH2O2 = (1).*0.01;   % control value of intracellular H2O2
cNADPH = 50;
gly = 1850;
ser = 468;
GAA = 10;
PE = 100;
GARP = 10;
AICARP = 2.1;
HCOOH = 500;
DUMP = 20;
b12 = p.val(p.b12); % VVMS factor for b12 deficiency
b6 = p.val(p.b6);
f = p.val(p.f); %folate

% methylation Vmaxes with male-female differences
p.val(p.gnmtv) = 0.7;
p.val(p.gamtv) = 1.3;

gnmtv = p.val(p.gnmtv);
othv = p.val(p.othv);
dnmtv = p.val(p.dnmtv);
gamtv = p.val(p.gamtv);
pemtv = p.val(p.pemtv);


ode_opt = {};
TIME = [0 5000];

p4 = p.val(p.p4);
e2 = p.val(p.e2);
```

```
% estradiol effect on PEMT and CBS
ppe = PEMTspline(e2);
ce = CBSspline(e2);

% estradiol effect on sphingomyelin
a1_sphmy = 2; % max for E2 fcn for Sphmy
a2_sphmy = 1.9652; %kd for E2 fcn for Sphmy
Sphmy = 10*(1+((a1_sphmy.*(e2-0.09))./(a2_sphmy + (e2-0.09))));


Initial = [3.36 1.05 12.42 0.02 0.56 0.79  15.22  17.95   3.52   1.03
0.0010 0.3211 0.6745 0 858.97 ...
 282.17  54.28  17.56 41.95 128.26   9.46 8393.34 127.71  13.22 0.9977
22.3832 41.0262 1847.7933];
[T,Y] = ode15s(@(t,y)msc(t,y,p),TIME,Initial,ode_opt);


% save the final concentrations and rates
mthf = Y(length(T),1);
thf  = Y(length(T),2);
fthf = Y(length(T),6);
dhf  = Y(length(T),3);
ch2 = Y(length(T),4);
ch  = Y(length(T),5);
met = Y(length(T),7);
sam = Y(length(T),8);
sah = Y(length(T),9);
hcy = Y(length(T),10);
gnmt = Y(length(T),11);
gnmt5mthf = Y(length(T),12);
mthfgnmt5mthf = Y(length(T),13);
folatefree = 20*f;
bet = Y(length(T),15);
betald = Y(length(T),16);
cho = Y(length(T),17);
pc = Y(length(T),18);
cyst = Y(length(T),19);
ccys = Y(length(T),20);
glutcys = Y(length(T),21);
cgsh = Y(length(T),22);
cgssg = Y(length(T),23);
bgsh = Y(length(T),24);
bgssg = Y(length(T),25);
bcys = Y(length(T),26);
bglut = Y(length(T),27);
cglut = Y(length(T),28);
cgly = gly;
cser = ser;





vdhfr = VDHFR(dhf,cNADPH,e2);
```

```
vts = VTS(DUMP,ch2,e2);
vmhd = VMHD(ch2,ch);
vmch = VMCH(ch,fthf);
vpgt = VPGT(fthf,GARP);
vart = VART(fthf,AICARP);
vfts = VFTS(thf,900,fthf);
vftd = VFTD(fthf);
vch2 = VCH2(thf,HCOOH,ch2);
vshmt = VSHMT(cser,thf,cgly,ch2,e2,b6);
vmthfr = VMTHFR(ch2,cNADPH,sam,sah,e2);
vvms = VVMS(mthf,hcy,e2,b12);
vbhmt = VBHMT(hcy,bet,sam,sah,e2,H2O2,ssH2O2);
vah = VAH(sah,hcy);
vdnmt = VDNMT(sam,sah,dnmtv);
vgnmt = VGNMT(sam,sah,mthf,cgly,gnmt,gnmt5mthf,gnmtv);
vgamt = VGAMT(sam,sah,GAA,gamtv);
vpemt = VPEMT(sam,sah,PE,ppe,pemtv);
vbah = VBAH(betald);
vcho = VCHO(cho);
vppl = VPPL(pc);
vsms =  VSMS(Sphmy);
voth = VOTH(sam,sah,othv);

vmatI = VMATI(met,sam);
vmatIII = VMATIII(met,sam);

vamd1 = VAMD1(sam);

folatesum = mthf+thf+dhf+ch2+ch+fthf + gnmt5mthf + 2*mthfgnmt5mthf;
sixsum = mthf+thf+dhf+ch2+ch+fthf;

total =(mthf+thf+dhf+ch2+ch+fthf);

transmeth = vgnmt+vdnmt+vgamt+vpemt+voth;
remeth = vvms+vbhmt;

vcbs = VCBS(hcy,sam,sah,bet,b6,ce,cser,H2O2,ssH2O2,cgsh);
vctgl = VCTGL(cyst);
vgcl = VGCL(ccys,cglut,cgsh,glutcys,H2O2,ssH2O2,e2);
vcysin = Vcysin(bcys,ccys);
vgs = VGS(cgly,glutcys,cgsh);
vgshout_h = VGSHout_h(cgsh);
vgshout_l = VGSHout_l(cgsh);
vgpx = VGPX(cgsh,H2O2,ssH2O2,e2,p4);
vgr = VGR(cgssg,cNADPH);
vgssgout_h = VGSSGout_h(cgssg);
vgssgout_l = VGSSGout_l(cgssg);
vglutin = Vglutin(cglut,bglut);
cysinb = cysin(T(end));
glutinb = glutin(T(end));

frac = vcbs/(vcbs+ vvms + vbhmt);
transsulf = vcbs;
    save(type);
```

```
end


%% utility functions used in parameter setup

function p = parameters

p.b12 = 1;
p.b6 = 2;
p.f = 3;
p.METIN = 4;
p.BETIN = 5;
p.e2 = 6;
p.gnmtv = 7;
p.othv = 8;
p.dnmtv = 9;
p.gamtv = 10;
p.pemtv = 11;
p.p4 = 12;

p.val(p.b12) = 1;
p.val(p.b6) = 1;
p.val(p.f) = 1;         % normal folate = 1
p.val(p.METIN) = 40;
p.val(p.BETIN) = 13;
p.val(p.e2) = 0.09;
p.val(p.p4) = 0.318;

% methylation Vmaxes for standard male , change all at once in main
program
p.val(p.gnmtv) = 0.3;
p.val(p.othv) = 1.4;
p.val(p.dnmtv) = 2;
p.val(p.gamtv) = 1.6;
p.val(p.pemtv) = 0.6;

end

function pemtActivity = PEMTspline(e2)
%{
Input:
    e2            estradiol
Output:
    pemtActivity  PEMT enzyme activity
%}

pemtActivity = 1 + 1.2904*e2./(1.0402+e2);

end

function cbsActivity = CBSspline(e2)
%{
Input:
    e2            estradiol
```

```
Output:
    cbsActivity   CBS enzyme activity
%}

cbsActivity = 1 + (e2-.09)./(.67+(e2-.09));

end



%% RHS of ordinary differential equations

function dy = msc(t,y,p)

dy=zeros(28,1);

GAA = 10;
k1 = 50;  % k1,k2,k3,k4 based on Kd values from Luka08 (and wagner)
k2 = 1;
k3 = 1;
k4 = 1.6;
n = 1;    % normal niacin = 1
PE = 100;
cholin = 200;
H2O2 = (1)*0.01; % change for oxidative stress experiments
ssH2O2 = (1).*0.01;   % control value of intracellular H2O2
cNADPH = 50;
gly = 1850;
ser = 468;





b12 = p.val(p.b12); % VVMS factor for b12 deficiency
b6 = p.val(p.b6); % factor for b6

betin = p.val(p.BETIN);

METIN = p.val(p.METIN);

% methylation Vmaxes with male-female differences
gnmtv = p.val(p.gnmtv);
othv = p.val(p.othv);
dnmtv = p.val(p.dnmtv);
gamtv = p.val(p.gamtv);
pemtv = p.val(p.pemtv);

%%%%%%%%%%
p4 = p.val(p.p4);
e2 = p.val(p.e2);

% estradiol effect on PEMT and CBS
ppe = PEMTspline(e2);
```

```
ce = CBSspline(e2);
%%%%%%%%%%

% Sphmy sex differences
a1_sphmy = 2; % max for E2 fcn for Sphmy
a2_sphmy = 1.9652; %kd for E2 fcn for Sphmy
Sphmy = 10*(1+((a1_sphmy.*(e2-0.09))./(a2_sphmy + (e2-0.09))));

%%%%%%%%%%

dy(1) =  (n.*VMTHFR(y(4),50,y(8),y(9),e2) - VVMS(y(1),y(10),e2,b12) +
k2.*y(12) - 2.*k1.*y(1).*y(11) + k4.*y(13) - k3.*y(1).*y(12));
dy(2) =  (VFTD(y(6)) +  VVMS(y(1),y(10),e2,b12) + VPGT(y(6),10) +
VART(y(6),2.1) - VFTS(y(2),900,y(6)) - VSHMT(ser, y(2),gly,y(4),e2,b6) -
VCH2(y(2),500,y(4))  + n.*VDHFR(y(3),50,e2));
dy(3) =  (VTS(20, y(4),e2) - n.*VDHFR(y(3), 50,e2));
dy(4) =  (VSHMT(ser, y(2),gly,y(4),e2,b6)  +  VCH2(y(2),500,y(4))  -
VTS(20, y(4),e2) - n.*VMTHFR(y(4),50,y(8),y(9),e2) - n.*VMHD(y(4),y(5)));
dy(5) =  (n.*VMHD(y(4),y(5)) - VMCH(y(5),y(6)));
dy(6) =  (VMCH(y(5),y(6)) + VFTS(y(2),900,y(6)) -  VPGT(y(6),10) -
VART(y(6),2.1) - VFTD(y(6)));
dy(7) =  (VBHMT(y(10),y(15),y(8),y(9),e2,H2O2,ssH2O2) +
VVMS(y(1),y(10),e2,b12) + metin(t,METIN) - VMATI(y(7),y(8)) -
VMATIII(y(7),y(8)));
dy(8) =  (VMATI(y(7),y(8)) +  VMATIII(y(7),y(8)) -
VGNMT(y(8),y(9),y(1),gly,y(11),y(12),gnmtv) - VDNMT(y(8),y(9),dnmtv) -
gab(t).*VGAMT(y(8),y(9),GAA,gamtv) - VPEMT(y(8),y(9),PE,ppe,pemtv) -
VOTH(y(8),y(9),othv) - VAMD1(y(8)));
dy(9) =  (VGNMT(y(8),y(9),y(1),gly,y(11),y(12),gnmtv) +
VDNMT(y(8),y(9),dnmtv) + gab(t).*VGAMT(y(8),y(9),GAA,gamtv)+
VPEMT(y(8),y(9),PE,ppe,pemtv) + VOTH(y(8),y(9),othv) - VAH(y(9),y(10)));
dy(10) = (VAH(y(9),y(10)) -
VCBS(y(10),y(8),y(9),y(15),b6,ce,ser,H2O2,ssH2O2,y(22)) -
VBHMT(y(10),y(15),y(8),y(9),e2,H2O2,ssH2O2) - VVMS(y(1),y(10),e2,b12));
dy(11) = (k2.*y(12) - 2.*k1.*y(1).*y(11));
dy(12) = (-k2.*y(12) + 2.*k1.*y(1).*y(11) - k3.*y(1).*y(12) + k4.*y(13));
dy(13) = (k3.*y(1).*y(12) - k4.*y(13));
dy(14) = sin(t);
dy(15) = (betin + VBAH(y(16)) -
VBHMT(y(10),y(15),y(8),y(9),e2,H2O2,ssH2O2) -.0096*y(15));
dy(16) = (VCHO(y(17)) - VBAH(y(16)) - .01*y(16));
dy(17) = (cholin + VPPL(y(18)) - VCHO(y(17)) - cholout(y(17)));
dy(18) = (VPEMT(y(8),y(9),PE,ppe,pemtv) + VSMS(Sphmy) - VPPL(y(18)));

dy(19) = (VCBS(y(10),y(8),y(9),y(15),b6,ce,ser,H2O2,ssH2O2,y(22)) -
VCTGL(y(19)));
dy(20) = (VCTGL(y(19)) - VGCL(y(20),y(28),y(22),y(21),H2O2,ssH2O2,e2) +
Vcysin(y(26),y(20)) - .35.*y(20).*y(20)./200);
dy(21) = (VGCL(y(20),y(28),y(22),y(21),H2O2,ssH2O2,e2) -
VGS(gly,y(21),y(22)));
dy(22) = (VGS(gly,y(21),y(22)) - VGSHout_h(y(22)) - VGSHout_l(y(22)) -
2.*VGPX(y(22),H2O2,ssH2O2,e2,p4) + 2.*VGR(y(23),cNADPH) - 0.002.*y(22));
dy(23) = (VGPX(y(22),H2O2,ssH2O2,e2,p4) - VGR(y(23),cNADPH) -
VGSSGout_h(y(23)) - VGSSGout_l(y(23)) - .1.*y(23));
```

```
dy(24) = (VGSHout_h(y(22)) + VGSHout_l(y(22)) - .10.*7.*y(24) -
.9.*100.*y(24));
dy(25) = (VGSSGout_h(y(23)) + VGSSGout_l(y(23)) - .10.*75.*y(25) -
.9.*75.*y(25));
dy(26) = (.9.*100.*y(24) + 2.*.9.*75.*y(25) + cysin(t) -
Vcysin(y(26),y(20)) - 0.35.*y(26));
dy(27) = (.9.*100.*y(24) + 2.*.9.*75.*y(25) + glutin(t) -
Vglutin(y(28),y(27)) -(.1).*y(27));
dy(28) = (Vglutin(y(28),y(27)) -
VGCL(y(20),y(28),y(22),y(21),H2O2,ssH2O2,e2) - (.07).*y(28));

% y(1) = 5mTHF
% y(2) = THF
% y(3) = DHF
% y(4) = 510CH2
% y(5) = 510CH
% y(6) = 10fTHF
% y(7) = met
% y(8) = sam
% y(9) = sah
% y(10) = hcy
% y(11) = GNMT
% y(12) = GNMT-5mTHF
% y(13) = 5mTHF-GNMT-5mTHF
% y(15) = betaine
% y(16) = bet-ald
% y(17) = cho
% y(18) = pc   Phosphocholine

% y(19) = cystathionine
% y(20) = cytosolic cysteine
% y(21) = glut-cys
% y(22) = cytosolic GSH
% y(23) = cytosolic GSSG
% y(24) = Blood GSH
% y(25) = Blood GSSG
% y(26) = Blood Cysteine
% y(27) = Blood Glutamate
% y(28) = Cytosolic Glutamate

end


%% reaction velocities

function a = VMTHFR(b,c,d,e,e2)
% b = 510CH2
% c = NADPH  (specified in main program)
% d = adomet
% e = adohcy
% e2 = estradiol

a1 = .9; % max for E2 fcn
a2 = 1.2138; %kd for E2 fcn
```

```
k1 =   50;   %August 8, 2005
k2 =   16;   %August 8, 2005
V  = (1-((a1.*(e2-0.09))./(a2 + (e2-0.09)))).*2000;  %female=0.8

a = (V.*b.*c./((k1+b).*(k2+c))).*(10./(10 + (d-e))).*((10 + 25.7)./10) ;
%long-range in

end


function a = VVMS(c,b,e2,b12)
% b = hcy
% c = 5mTHF
% e2 = estradiol
% b12 = effect of b12 deficiency


a1 = 2; % max for E2 fcn
a2 = 1.6349; %kd for E2 fcn

k12 = (1).*b12.*(1).*(1+((a1.*(e2-0.09))./(a2 + (e2-
0.09)))).*(.5).*(1.2)*406;
k13 = 25;
k14 =  1;

a = (1)*k12.*((b./k14)./(1 + (b./k14))).*(c./k13)./(1 + (c./k13));

end

% b = 10fTHF


function a = VFTD(b)

k1 =   20;
V  =  500;

a = V.*(b./k1)./(1 + (b./k1)) ;
end


function a = VPGT(b,c)

% b = 10fTHF
% c =  GARP  (needs to be specified in main program)

k1 =   4.9 ;
k2 =    520 ;
V  =   24300 ;

a = V.*(b./k1).*(c./k2)./(1 + (b./k1) + (c./k2) +  (b./k1).*(c./k2));
end
```

```
function a = VART(b,c)
% b = 10fTHF
% c = A ICARP  (needs to be specified in main program)


k1 =   5.9;  %Km10fTHF
k2 =    100 ;   %KmAICARP
V  =   (1)*55000 ;

a = V.*(b./k1).*(c./k2)./(1 + (b./k1) + (c./k2) +  (b./k1).*(c./k2));
end




function a = VFTS(b,c,d)
% b = THF
% c =  HCOOH  (needs to be specified in main program)

k1 =   3;    % was 10  KmTHF
k2 =   43;    %KmHCOOH
V  =   (1)*3900;

a = V.*(b./k1).*(c./k2)./(1 + (b./k1) + (c./k2) +  (b./k1).*(c./k2));
end



function a = VSHMT(b,c,d,e,e2,b6)
% b = Serine  (specified in main program)
% c = THF
% d = Glycine (specified in main program)
% e = 510CH2
% f = free SHMT
% positive direction from THF towards 510CH2
% e2 = estradiol

k1  = 600;  %Km for ser
k2  = 50;   %Km for THF

a1 = 2; % max for E2 fcn
a2 = 0.2312; %kd for E2 fcn
V1 = b6*(1+((a1.*(e2-0.09))./(a2 + (e2-0.09))))*40000;

k3 = 3000;  %Km for gly
k4 = 3200;  %Km for ch2
V2 = b6*(1+((a1.*(e2-0.09))./(a2 + (e2-0.09))))*2500000;

a = V1.*(b./k1).*(c./k2)./(1+(b./k1)+(c./k2)+(b./k1).*(c./k2))...
    -V2.*(d./k3).*(e./k4)./(1+(d./k3)+(e./k4)+(d./k3).*(e./k4));

end
```

```
function a = VCH2(b,c,d)
% b = THF
% c = HCOOH (must be specified in main program)
% d = 510CH2

k1 = 0.15;
k2  = 12;

a = k1.*b.*c  -  k2.*d;
end


function a = VDHFR(b,c,e2)
% b = DHF
% c = NADPH  (needs to be specified in main program)
% e2 = estradiol

k1 =   0.5 ;  %KmDHF
k2 =    4.0 ;   %KmNADPH
V  =   (100)*50;

a = (1+2.*(e2)./(19+(e2))).*V.*(b./k1).*(c./k2)./(1 + (b./k1) + (c./k2) +
(b./k1).*(c./k2));

end


function a = VTS(b,c,e2)
% b = DUMP (needs to be specified in main program)
% c =  510CH2
% e2 = estradiol

k1 =   6.3 ;  %KmDUMP
k2 =   14;   %Km510CH2
V  = (1)*(100)*50;

a = (1+2*(e2)./(19+(e2)))*V.*(b./k1).*(c./k2)./(1 + (b./k1) + (c./k2) +
(b./k1).*(c./k2));

end


function a = VMHD(b,c);
%  b = 510CH2
%  c  = 510CH

k1 = 2;
V1 = (1)*200000;
k2 = 10;
V2 = (1)*594000;


a =  V1.*(b./k1)./(1 + b./k1)  - V2.*(c./k2)./(1 + c./k2);
end
```

```
function a = VMCH(b,c);
% b = 510CH
% c = 10fTHF

k1 = 250;
V1 = (1)*800000;
k2 = 100;
V2 = (1)*20000;


a =  V1.*(b./k1)./(1 + b./k1)  - V2.*(c./k2)./(1 + c./k2);
end



function a = VBHMT(h,b,d,c,e2,h2o2,ssh2o2)
% b = betaine
% h = hcy
% d = adomet
% c = adohcy
% e2 = estradiol
% h2o2 = cytosolic H2O2 (specified in main program)
%ssh2o2 = steady state H2O2 (specified in main program)


a1 = 0.9; % max for E2 fcn
a2 = 0.4335; %kd for E2 fcn
k17 = (1-((a1.*(e2-0.09))./(a2 + (e2-0.09)))).*2000;
k18 = 12;  %Km for Hcy
k19 = 2000;
ki=.01;


a =  (.8)*(k17.*h.*b./((k18+h).*(k19+b))).*(exp(-.0021.*(c +
d))).*(exp(+.0021.*(28.75))).* ...
    (1 + (.16).*(b-346.61)/346.61)... %betaine effect
   .* ((ssh2o2+ki)./(h2o2+ki));

end

function f=metin(t,p)
%p = METIN
f = p;
end

function a = VMATI(b,c);

%c = adomet concentration
%b = methionine concentration

k1 = 260;  %650;    %VMATImax (activity)
```

```
k2 = 41;   %41;     %VMATIm (Mich const for Met)

a = (1)*(0.23 + 0.8.*exp(-0.0026.*c)).*k1.*(b./k2)./(1 + (b./k2));

end

function a = VMATIII(b,c);

%c = adomet concentration
%b = methionine concentration

k1 = 220;   %(1)*220; %100;    %VMATImax (activity)
k2 = 300;  %301.3;     %VMATIm (Mich const for Met)
k3 = 360*360;     %VMATIi (inhib for adomet)

a = k1.*(b.^(1.21)./(k2 + b.^(1.21)).*(1 +7.2.*c.^(2)./(k3 + c.^(2)))) ;
end


function a = VGNMT(b,c,d,e,g,fg,gnmtv)
%b = sam
%c = sah
%d = 5mTHF
%e = glycine
%g = gnmt
%fg = gnmt-5mTHF


k2 = 100;    %Km for SAM Clarke and Banfield Hcy
k3 = 35;     %Ki for SAH, HCY
k4 = 12.2;   %KM glycine
k1 = (gnmtv)*(1750);

a = k1.*(g + (.5).*fg).*(b./((k2.*(1 + c./35)) + b));
end


function a = VDNMT(b,c,dnmtv)

%b = adomet
%c = adohcy

k66 = (1).*(dnmtv)*(2.5);
k77 = 1.4;
k78 = 1.4;

a =  k66.*b./(k77.*(1 + c./k78) + b);
end

function f=gab(t);
n=length(t);
for i=1:n
f(i) =1;
end
```

```
end

function a = VGAMT(b,c,d,gamtv);

%b = SAM
%c = SAH
%d= GAA (specified in main program)

k1 = (gamtv).*(90);    %Vmax
k2 = 49;    % Km for SAM
k3 = 16;    % Ki ofr SAH

a = k1.*(b./(k2.*(1 + c./k3) + b));
end



function a = VPEMT(b,c,d,fe,pemtv)
%b = sam
%c = sah
%d = pe
% fe = pemt fold dependent on e2

k1 = (1).*pemtv*fe*98;   %Vmax

k2 = 18.2;  % Km for SAM
k3 = 3.8;   %Ki for SAH
k4 = 5000;     %Km for PE
d=100;

a = k1.*((b./(k2 + b))).*(1./(1 + c./k3)).*(25*d/(k4+d));
end



function a = VOTH(b,c,othv)

%b = SAM
%c = SAH

k1 = (1).*(othv)*(40);
k2 = 50;
k3 = 15;

a = k1.*b./(k2.*(1 + c./k3) + b);
end


function a = VAMD1(b)
% b = sam

  Km = 245;
  Vmax = 100;
```

```
  a = Vmax.*b./(Km+b);

end


function a = VAH(b,c);

%b = adohcy concentration
%c = hcy concentration

k20 = (1)*320;
k21 = 6.5;
k22 = 150;
k200 = 755;


a = (1)*k20.*(b./k21)./(1 + (b./k21)) - (6)*k200.*(c./k22)./(1 +
(c./k22)) ;
end


function a = VCBS(b,d,e,f,b6,fe,g,h2o2,ssh2o2,gsh)
% b= hcy
% d = adomet
% e = adoHcy
% f = betaine
% fe = cbs fold dependent on e2
% g = cytosolic serine
% h2o2 = cytosolic h2o2 (specified in main program)
% ssh2o2 = steady state h2o2 (specified in main program)
% gsh = cgsh

k30 = (1).*fe*b6*29*170/4.2;
k31 = 170;
k32 = 2000;
ka = 0.035;

Vmax_gsh = .4;
Km_gsh = 6000;
Hill_gsh = 10;

GSHFcn = (1+((Vmax_gsh.*gsh.^Hill_gsh)./(Km_gsh.^Hill_gsh +
gsh.^Hill_gsh)));

a = (.75).*k30.*b./(k31+b) .* (4.6).*(d+e)./(14+(d+e))...
    .*(1 + (1).*H1(f-346.61).*(.25).*(f-346.61)./(100 + (f-
346.61))).*((h2o2+ka)./(ssh2o2+ka)).*GSHFcn;
end

function a = VBAH(b);

%b = betaine aldehyde

k17 = 45;
```

```
k18 = 250;  %Km for betaine aldehyde


a =  k17.*b./(k18 + b);
end

function a = VCHO(b);

%b = choline

k17 = 125;
k18 = 200;  %Km for choline

a =  k17.*b./(k18 + b);
end

function a = VPPL(b);

%b = pc

k17 = 525;
k18 = 400;  %Km for phosphocholine

a =  k17.*b./(k18 + b);
end



function a = cholout(c);
%c = choline

k1 = (.97)*220;   %Vmax
k2 = 5;  % Km


a = k1.*(c./(k2 + c));

end

function a = VSMS(b);

%b = sphmy

k17 = 30;
k18 = 20;  %Km for phosphocholine

a =  k17.*b./(k18 + b);
end


function a = VCTGL(b);
% b = cystathionine

k1 = 500;
```

```
V = (1./3.5).*1500;

a = V.*(b./k1)./(1 + (b./k1));
end



function a = VGCL(b,c,d,e,h,s,e2);
% b = cysteine
% c = glutamate (needs to be set in main program)
% d = glutathione
% e = glu-cys
%h = H2O2
%s = ssH2O2
%e2 = estradiol


 V = .8*4500;

k1 = 100;       %Km cys
k2 = 1900;      %Km glut
k3 = 5597;
k_a = .01;
ki = 8200;
kp = 300;


a = (1 + 25.7716.*(e2-0.09)./(10.8 + (e2-0.09))).*(V.*(c.*b -
e./k3)./((k1.*k2 + k1.*c + b.*k2.*(1 + d./ki + c./k2) + e./kp +
d./ki))).*(k_a + h)./(k_a + s);
end



function a = Vcysin(b,c)
%b = blood cysteine
%c = cytosolic cysteine

k1 = (9).*14950;   %vmax
k2 = 2100;
k3 = 0.25;

a = k1.*b./(k2 + b) - k3.*c;
end


function a = VGS(b,c,d);
% b = glycine (constant) needs to be set in main program
% c = glucys
% d = gsh


k1 = 22;  % km for glucys
```

```
k2 = 300; % km for glycine
kp  = 30; % dissoc. const for GSH
keq = 5600; %equil const for overall reaction
V = (1).*5400;

a = V.*(b.*c - d./keq)./(k1.*k2 + c.*k2 + b.*k1.*(1 + c./k1) + d./kp);
end

function a = VGSHout_h(b);

km = 150;
vmax = 150;

a = (vmax .* b)./(km + b);
end

function a = VGSHout_l(b);

km = 3000;
vmax = 1100;

a = (vmax .* b.*b.*b)./ ((km)^3 + b.*b.*b);
end


function a = VGPX(gsh, H2O2, ssH2O2,e2,p4);

km_g = 1330;
km_h = ssH2O2;
vmax = .8*4500;
p4_effect = (1 - 0.3.*(p4-0.318)./(70 + (p4-0.318))); %p4 effect on gpx
e2_effect = (1 + 27.*(e2-0.09)./(11 + (e2-0.09))); % e2 effect on gpx

a = p4_effect.*e2_effect.*vmax.* ((gsh./(km_g + gsh)).^2) .*
(H2O2./(9.*km_h + H2O2));
end

function a = VGR(gssg, cNADPH);

km1 = 107;        % for gssg
km2 = 10.4;       % for NADPH

vmax = (1)*892.5;

a = (vmax .* (gssg ./ km1) .* (cNADPH ./ km2)) ./ (1 + gssg./km1 +
cNADPH./km2 + (gssg./km1).*(cNADPH./km2));
end

function a = VGSSGout_h(b);

km = 1250;
vmax = 40;
a = (vmax .* b)./(km + b);
end
```

```
function a = VGSSGout_l(b);

km = 7100;
vmax = 4025;

a = (vmax .* b)./(km + b);
end

function f= cysin(t);
n=length(t);
for i=1:n
    f(i) = (1)*70;
end
end

function f= glutin(t);
n=length(t);
for i=1:n
    f(i) = (1)*200;
end
end



function a = Vglutin(c,b);
%c = cytoplasmic glut
%b = blood glut

k1 = 28000;  %Vmax in
k2 = 300;   %Km in
k3 = 1;   % mass action rate out

a = k1.*b./(k2 + b) - k3.*c;
end

function f=H1(a)
%used in CBS function
if a < 0
      f = 0;
else
    f = 1;

end
end
```
